# Head nurses’ scientific research and innovation leadership and nurses’ innovative work behavior: the mediating role of intra-team competition perception and the moderating role of harmonious work passion

**DOI:** 10.3389/fpsyg.2026.1900030

**Published:** 2026-09-09

**Authors:** Weiwei Yang, Simin Zhu, Junjie Xu, Linxin Xie, Yuting Li, Hongzhen Xie

**Affiliations:** 1 School of Nursing, Guangdong Pharmaceutical University, Guangzhou, China; 2 Health Science Center, Yangtze University, Jingzhou, China; 3 School of Nursing, Southern Medical University, Guangzhou, China; 4 Department of Health Medicine, General Hospital of Southern Theater Command of PLA, Guangzhou, China

**Keywords:** harmonious work passion, head nurses, innovative work behavior, intra-team competition perception, moderated mediation, nurses, nursing leadership, research-innovation leadership

## Abstract

**Background:**

Nurses’ innovative work behavior represents an important form of proactive workplace behavior in healthcare organizations. Although head nurses’ scientific research and innovation leadership may create conditions that support innovation, how such leadership is translated into nurses’ innovative work behavior through team-level cognitive processes and under what motivational conditions this association is strengthened or weakened remain insufficiently understood.

**Methods:**

A cross-sectional survey was conducted among 400 clinical nurses in China. Participants completed validated measures of head nurses’ scientific research and innovation leadership, intra-team competition perception, harmonious work passion, and nurses’ innovative work behavior. Pearson correlation analyses, hierarchical regression analysis, mediation analysis, and moderated mediation analysis were performed.

**Results:**

Head nurses’ scientific research and innovation leadership was positively associated with nurses’ innovative work behavior. Intra-team competition perception partially mediated this association. Harmonious work passion moderated the association between intra-team competition perception and nurses’ innovative work behavior: the association was stronger among nurses with lower harmonious work passion and weaker among those with higher harmonious work passion. The conditional indirect association between head nurses’ scientific research and innovation leadership and nurses’ innovative work behavior through intra-team competition perception was also stronger when harmonious work passion was lower.

**Conclusion:**

By integrating transformational leadership theory and self-determination theory, this study extends existing research on nurses’ innovative work behavior by identifying intra-team competition perception as a team-level cognitive pathway and harmonious work passion as a motivational boundary condition linking leadership context with innovation behavior. Nursing managers should not rely solely on competitive cues to stimulate innovation, but should also cultivate nurses’ internalized work passion and provide supportive leadership for sustainable innovation.

## 1 Introduction

In the context of continuous advances in medical technology and ongoing optimization of nursing services, improvements in nursing quality increasingly rely on the innovative practices of frontline nurses (Masood and Afsar, 2017). As frontline managers maintaining the most direct engagement with clinical nursing staff, head nurses’ scientific research and innovation leadership is considered an important factor associated with individual innovative behavior and the overarching team innovation climate (Wang et al., 2025). Previous studies have extensively examined nurses’ innovative work behavior from the perspectives of individual traits, organizational support, and leadership factors (Almutairi et al., 2025; Labrague and Toquero, 2023). Consistent with broader organizational research, meta-analytic evidence has demonstrated that leadership factors have been identified as important correlates of creativity and innovation through their associations with employees’ motivation and work environments (Lee et al., 2020). However, several important gaps remain. First, the role of leadership specifically oriented toward scientific research and innovation in nursing contexts has received limited attention compared with general leadership styles. Second, the psychological mechanisms and motivational conditions underlying the association between leadership and nurses’ innovative work behavior remain insufficiently understood. Transformational leadership theory, originally introduced by Burns (1978) and further developed by Bass (1985), proposes that leaders motivate followers to transcend basic role expectations and engage in proactive and innovative behaviors. Bass (1994) further conceptualized transformational leadership as comprising four dimensions: idealized influence, inspirational motivation, intellectual stimulation, and individualized consideration. Through these mechanisms, transformational leadership is theoretically expected to be associated with an innovation-conducive work environment and higher levels of members’ innovative behaviors (Corthésy-Blondin et al., 2025; Zhang et al., 2022). In nursing contexts, head nurses’ scientific research and innovation leadership can be understood as reflecting transformational leadership mechanisms in the nursing context, as it emphasizes innovation-oriented vision, intellectual stimulation, and support for nurses’ creative efforts. However, this theory remains relatively limited in explaining why individuals in the same leadership context may exhibit different levels of innovative behavior (Lee and Seo, 2024). Self-determination theory further posits that behavioral engagement is governed not merely by exogenous environmental factors, but by intrinsic motivation and the degree to which the fundamental psychological needs for autonomy, competence, and relatedness are fulfilled (Ryan and Deci, 2020). Consequently, the present study adopts a “leadership context–team cognition–individual behavior” framework to examine how head nurses’ scientific research and innovation leadership is associated with nurses’ innovative work behavior through intra-team competition perception. In addition, this study examines whether harmonious work passion moderates the association between intra-team competition perception and innovative work behavior. By integrating transformational leadership theory and self-determination theory, this study aims to clarify a team-based psychological pathway and an intrinsic motivational boundary condition underlying nurses’ innovative work behavior.

## 2 Background

Innovative work behavior refers to employees’ behaviors involving the generation, promotion, and realization of new ideas within the workplace (Scott and Bruce, 1994). Later studies further conceptualized innovative work behavior as a multi-stage process involving idea generation, idea promotion, and idea implementation (De Jong and Den Hartog, 2010). In nursing contexts, such behaviors reflect nurses’ proactive efforts to develop and implement novel approaches to improve healthcare practices. This behavior depends not only on individuals’ proactive engagement but is also associated with organizational context and leadership factors (Liu et al., 2026; Zhang et al., 2022). As the most direct frontline leaders within nursing teams, head nurses’ scientific research and innovation leadership refers to their comprehensive capability to lead scientific research and innovation activities, integrate innovation resources, and stimulate team members’ participation in innovation within their nursing teams (Boamah et al., 2018). Although head nurses’ scientific research and innovation leadership shares conceptual similarities with transformational leadership in terms of vision guidance, intellectual stimulation, and innovation support, it represents a context-specific leadership resource embedded in nursing research and innovation activities. Unlike general leadership styles, scientific research and innovation leadership emphasizes research guidance, innovation resource integration, and the facilitation of nurses’ participation in innovation practices (Lin, 2023). However, even under similar leadership contexts, nurses may still differ in their innovation attitudes and innovation engagement. Self-determination theory further posits that when individuals experience heightened levels of autonomy, competence, and relatedness within the occupational environment, they are more inclined to internalize external organizational demands into stable, sustained behavioral engagement (Hindman et al., 2026).

Previous studies have examined various pathways linking leadership to innovative behavior; however, less attention has been given to how nurses interpret leadership-related signals within their immediate team environments. On this basis, the present study introduces intra-team competition perception as a key mechanistic variable. Intra-team competition perception provides a team-level cognitive perspective by capturing nurses’ perceptions of performance comparison, developmental opportunities, and resource competition within teams (Brown et al., 1998). It may represent an important psychological pathway associated with the relationship between head nurses’ scientific research and innovation leadership and nurses’ innovative work behavior. Importantly, competition itself is not inherently beneficial or detrimental; rather, its consequences depend on how individuals interpret competitive cues and the surrounding team context (Deutsch, 1949; Fletcher et al., 2008). While destructive forms of competition may trigger negative interpersonal processes, such as knowledge hiding and reduced collaboration (Černe et al., 2014), competition may also function constructively when ompetitive environments are interpreted as task-oriented challenges that direct individuals’ attention toward achievement and performance improvement (Fletcher et al., 2008). Therefore, the present study does not assume that all forms of intra-team competition are beneficial; rather, its effects may vary depending on the leadership context and team climate, and under conditions of strong head nurses’ scientific research and innovation leadership, nurses may perceive intra-team competition as more closely associated with innovation-related activities and performance expectations (Černe et al., 2014). On this basis, head nurses may make nurses’ judgments about whether innovation is important and worth investing in more explicit by providing innovation support, strengthening innovation-related feedback, and fostering supportive team relationships, which may be associated with nurses’ greater tendency to propose new ideas, seek support, and promote the implementation of innovative behavior (Duan et al., 2025). According to self-determination theory, whether external contextual cues can be further transformed into sustained innovation engagement depends on whether individuals interpret them as signals that are meaningful and valuable. When the team context is perceived by nurses as more positive, supportive, and growth-promoting, they are more likely to be associated with proactive innovation engagement (Lee and Seo, 2024).

However, the impact of intra-team competition perception may exhibit variability across the nursing workforce. Harmonious work passion represents an individual’s positive affective investment in their professional activities and serves as a manifestation of stable intrinsic motivation (Vallerand et al., 2003). For nurses with lower levels of harmonious work passion, innovation goals are less likely to be internalized as stable self-requirements, and their innovation engagement is therefore more easily influenced by external contextual cues. In this case, the performance comparisons and developmental opportunities conveyed through intra-team competition perception may be more closely associated with nurses’ engagement in innovative work behavior (Ryan and Deci, 2020; Van den Broeck et al., 2016). In contrast, for nurses with higher levels of harmonious work passion, nursing values and continuous improvement are more likely to have already been internalized as self-requirements, and their innovative behavior is therefore relatively less dependent on external competitive cues. Therefore, the association between intra-team competition perception and nurses’ innovative work behavior may be weaker at higher levels of harmonious work passion.

**Figure 1:**
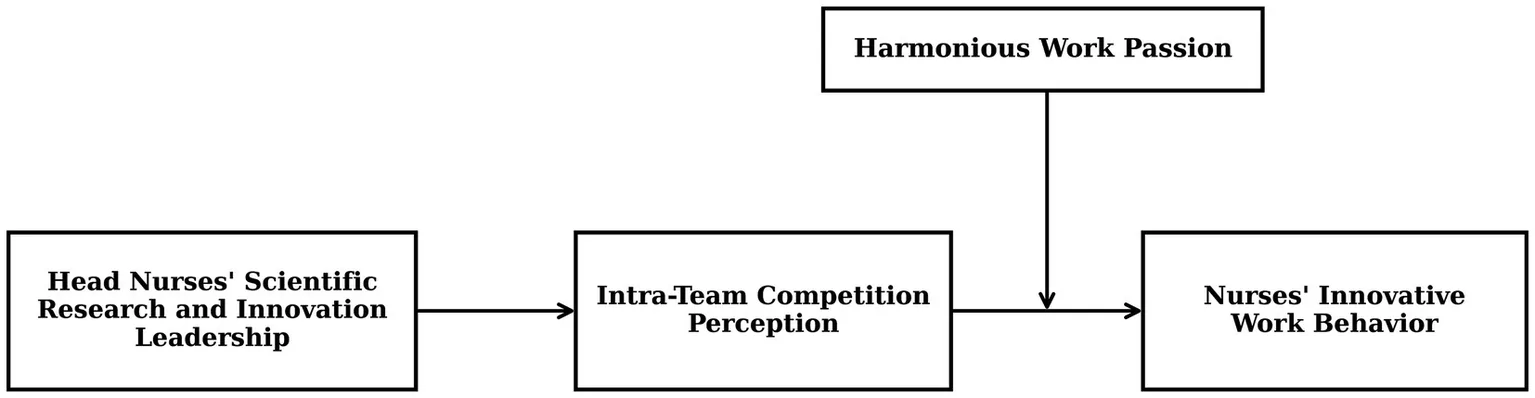
The theoretical framework of this study.

In brief, this study develops a mediation model in which head nurses’ scientific research and innovation leadership is indirectly associated with nurses’ innovative work behavior through intra-team competition perception, and further examines the moderating role of harmonious work passion. The proposed theoretical framework is shown in Figure 1.

## 3 The study

### 3.1 Aim

The present study aimed to examine the association between head nurses’ scientific research and innovation leadership and nurses’ innovative work behavior by developing a moderated mediation model. Specifically, this study sought to investigate whether intra-team competition perception serves as a mediating mechanism linking head nurses’ scientific research and innovation leadership with nurses’ innovative work behavior. In addition, this study examined whether harmonious work passion functions as a boundary condition influencing the association between intra-team competition perception and nurses’ innovative work behavior, as well as the indirect association between leadership and innovative behavior.

### 3.2 Hypotheses

Based on transformational leadership theory and self-determination theory, the present study proposes that head nurses’ scientific research and innovation leadership may be associated with nurses’ innovative work behavior through team-level cognitive processes and that this association may vary depending on nurses’ motivational characteristics. Specifically, intra-team competition perception is proposed as the mediating mechanism linking leadership context with innovative behavior, whereas harmonious work passion is proposed as a motivational boundary condition potentially conditioning this association.

*Hypothesis 1*: Head nurses’ scientific research and innovation leadership is positively associated with nurses’ innovative work behavior.

*Hypothesis 2*: Intra-team competition perception mediates the relationship between head nurses’ scientific research and innovation leadership and nurses’ innovative work behavior.

*Hypothesis 3*: Harmonious work passion moderates the association between intra-team competition perception and nurses’ innovative work behavior, such that this association is stronger when harmonious work passion is lower and weaker when harmonious work passion is higher.

*Hypothesis 4*: Harmonious work passion moderates the indirect association between head nurses’ scientific research and innovation leadership and nurses’ innovative work behavior through intra-team competition perception, such that the indirect association is stronger when harmonious work passion is lower and weaker when harmonious work passion is higher.

## 4 Methods

The STROBE reporting checklist (2019) was strictly adhered to in this cross-sectional study, thereby ensuring the transparency and reliability of the research findings.

### 4.1 Study design and sample

A cross-sectional survey was conducted among clinical nurses in China in September 2025 (Cuschieri, 2019). Participants were recruited using convenience sampling from 48 public tertiary hospitals located across five major geographical regions of China, including South China, East China, Central China, Southwest China, and North China. The survey link was distributed by nursing department directors to head nurses, who subsequently invited eligible clinical nurses within their departments to participate. The sample size was estimated using Kendall’s rule of thumb, which recommends 5–10 participants per questionnaire item. As the questionnaire contained 42 items, the required sample size was estimated to range from 210 to 420 participants. After allowing for a 25% invalid response rate, the target sample size was 263–525 participants. Ultimately, 400 valid questionnaires were included in the final analysis. To further strengthen the methodological rigor of the sample size estimation, an *a priori* power analysis was conducted using G*Power 3.1 (Faul et al., 2009) for hierarchical multiple regression analysis. Assuming a medium effect size (f^2^ = 0.15), an alpha level of 0.05, a statistical power of 0.90, and seven predictors, the minimum required sample size was approximately 130 participants. The inclusion criteria were: (1) registered nurses with a valid nursing license; (2) at least 1 year of clinical work experience; and (3) currently employed and on duty during the survey period. Head nurses, nursing managers, and nurses who were unable to complete the questionnaire during the survey period were excluded.

### 4.2 Data collection

Data were collected using Wenjuanxing, a widely used online survey platform in China. A total of 410 electronic questionnaires were distributed. The introductory page explained the study purpose, voluntary nature of participation, anonymity, and confidentiality. To improve data quality, all items were set as mandatory. In addition, Wenjuanxing was configured to reduce duplicate submissions by restricting repeated access from the same IP address and preventing multiple submissions from the same WeChat account. A total of 405 questionnaires were returned. Responses were excluded if they showed identical answers across all items, systematic response patterns such as repeated numerical sequences, or completion times shorter than 2 min. After screening, 400 valid questionnaires were retained, yielding an effective response rate of 97.56%.

### 4.3 Measures

All instruments used in this study were established scales with previously demonstrated psychometric properties. These instruments were adopted without modification, and their internal consistency reliability was assessed in the current sample.

#### 4.3.1 General information survey

In line with the study’s aims, the survey was created by the investigators and incorporated variables such as gender, age, professional title, and years of work experience.

#### 4.3.2 Head nurses’ scientific research and innovation leadership scale

This study used a specialized scale developed by Ma et al. (2023) to assess head nurses’ leadership in scientific research and innovation. The scale has 15 items covering five dimensions: foresight, charisma, influence, decisiveness, and control, with three items for each dimension. It uses a five-point Likert scoring method, with responses ranging from “strongly disagree” or “never” to “strongly agree” or “always,” scored from 0 to 4, yielding a total score ranging from 0 to 60. Higher scores indicate stronger leadership. The Cronbach’s α coefficient of this scale in the present sample was 0.951.

#### 4.3.3 Intra-team competition perception scale

The unidimensional scale developed by Zhou (2020) was adopted by this study to measure intra-team competition perception. The scale consists of three items and uses a five-point Likert scoring method, with answers from “strongly not true” to “strongly true,” scored from 1 to 5, with higher scores showing stronger feelings about competition between team members. A Cronbach’s α coefficient of 0.767 was obtained for this scale in the present sample.

#### 4.3.4 Nurses’ innovative work behavior scale

In order to evaluate the extent to which nurses exhibit innovative behavior, this study employed the three-dimensional scale that was developed by Bao et al. (2012). The scale includes 10 items. These cover three dimensions. These are idea generation, obtaining support and idea implementation. It uses something called a five-point Likert scoring method, where you can choose from “never,” “sometimes,” “often,” and “always.” The scores are from 1 to 5, and the total score ranges from 10 to 50. Elevated results are indicative of a superior degree of innovation. The Cronbach’s α coefficient for this study was 0.840.

#### 4.3.5 Harmonious work passion scale

To evaluate workers’ Harmonious Work Passion, this investigation chose the Harmonious Work Passion part of the Passion Scale created by Vallerand et al. (2003). The scale consists of seven items. It uses a seven-point Likert scoring method. Responses range from “completely inconsistent” to “completely consistent.” Scores range from 1 to 7. Higher scores indicate a higher level of Harmonious Work Passion. In this investigation, the Cronbach’s α coefficient was 0.844.

### 4.4 Data analysis

We used descriptive statistics, like frequencies, percentages, means and standard deviations, to sum up the study variables. These were head nurses’ scientific research and innovation leadership, nurses’ innovative work behavior, how they saw intra-team competition, and their passion for harmonious work. Preliminary analyses, including independent-samples *t*-tests and one-way analysis of variance (ANOVA), were conducted to examine the influence of demographic characteristics on these variables, while Pearson correlation analysis was used to explore inter-variable associations. To assess potential common method bias, Harman’s single-factor test was applied. Hypothesis testing was conducted using hierarchical regression and PROCESS macro analyses. Prior to conducting hierarchical regression analyses, the assumptions underlying regression models, including normality, multicollinearity, homoscedasticity, and outlier diagnostics, were assessed. No violations of these assumptions were identified. Multicollinearity was evaluated using tolerance values and variance inflation factors (VIFs). Following commonly recommended criteria, tolerance values above 0.10 and VIF values substantially below 5 indicate acceptable levels of multicollinearity (Hair et al., 2019). In the regression models, demographic variables (gender, age, and years of working) were entered in the first step as control variables. Subsequent steps involved the sequential entry of head nurses’ scientific research and innovation leadership, intra-team competition perception, and harmonious work passion. The final step incorporated the interaction term between intra-team competition perception and harmonious work passion to examine the moderating role of harmonious work passion. Furthermore, Models 4 and 14 of the PROCESS macro (version 4.1) were utilized to rigorously test the mediating role of intra-team competition perception and the moderated mediation effect of harmonious work passion, respectively. Significance was determined using bootstrapping with 5,000 resamples; associations or indirect estimates were considered statistically significant if the 95% confidence intervals (CIs) excluded zero and *p*-values were less than 0.05.

### 4.5 Ethical considerations

The study protocol was approved by the Ethics Committee of the Southern Theater Command General Hospital of the People’s Liberation Army (Approval No. NZLLKZ2025060). All procedures involving human participants were conducted in accordance with the Declaration of Helsinki and relevant institutional and national regulations. Before completing the online questionnaire, participants were informed of the study objectives, procedures, confidentiality, and voluntary nature of participation. Electronic informed consent was obtained through an informed consent statement presented at the beginning of the questionnaire.

## 5 Results

### 5.1 General information questionnaire

The demographic characteristics of the participants and the results of the univariate analyses of nurses’ innovative work behavior are presented in Table 1. Significant differences in nurses’ innovative work behavior were observed according to gender, age, years of work experience, and education level, with the corresponding effect sizes also reported in Table 1.

**Table 1:** Demographic characteristics and univariate analysis of nurses’ innovative work behavior (*N* = 400).

Variables	Category	*N* (%)	NIB score (M ± SD)	*t/F*	*p*	Effect size
Gender	Male	73 (18.2)	35.47 ± 9.80	3.372	0.001	−0.437
	Female	327 (81.8)	39.06 ± 7.85			
Age	30	140 (35.0)	36.02 ± 8.60	15.643	0.001	0.215
	30 ~ 40	139 (34.8)	37.72 ± 8.88			
	41	121 (30.3)	41.66 ± 6.15			
Years of working	3	92 (23.0)	33.25 ± 8.59	8.313	0.001	0.149
	3 ~ 5	81 (20.3)	38.27 ± 8.40			
	6 ~ 9	165 (41.3)	38.76 ± 8.19			
	10 ~ 15	18 (4.5)	41.33 ± 7.13			
	16	44 (11.0)	43.23 ± 4.47			
Education level	College	94 (23.5)	37.11 ± 8.75	9.779	0.001	0.047
	Undergraduate	277 (69.3)	38.19 ± 8.32			
	Graduate	29 (7.3)	44.66 ± 3.09			

NIB, nurses’ innovative work behavior; *t*, independent-samples *t*-test; *F*, one-way analysis of variance. Effect sizes are presented as Cohen’s *d* for gender and eta-squared (η^2^) for age, years of working, and education level.

### 5.2 Common method bias test

Harman’s single-factor test was performed to assess the potential for common method bias due to the self-report methodology. The results showed that the first principal factor accounted for 36.74% of the total variance. As this value is below the recommended threshold of 40% (Podsakoff et al., 2003), the results suggested that no single factor dominated the variance structure. However, Harman’s single-factor test alone cannot completely exclude the possibility of common method variance.

### 5.3 Correlation analysis among variables

The inter-correlations among the study variables are presented in Table 2, providing preliminary empirical support for the proposed hypotheses. Specifically, HNSRIL was positively and significantly associated with ITCP (*r* = 0.53, *p* < 0.001), HWP (*r* = 0.50, *p* < 0.001) and NIB (*r* = 0.55, *p* < 0.001). Furthermore, ITCP exhibited a positive correlation with NIB (*r* = 0.51, *p* < 0.001). Additionally, HWP demonstrated significant positive correlations with both ITCP (*r* = 0.41, *p* < 0.001) and NIB (*r* = 0.53, *p* < 0.001).

**Table 2:** Correlation analysis.

Variable	M	SD	1	2	3	4
1. HNSRIL	46.81	12.03	–			
2. ITCP	11.17	3.30	0.53***	1		
3. HWP	34.00	9.81	0.50***	0.41***	1	
4. NIB	38.41	8.34	0.55***	0.51***	0.53***	1

M, mean; SD, standard deviation; HNSRIL, Head nurses’ scientific research and innovation leadership; ITCP, Intra-team competition perception; HWP, Harmonious work passion; NIB, nurses’ innovative work behavior; ***p < 0.001.

### 5.4 Multicollinearity assessment

Before testing the proposed hypotheses, multicollinearity diagnostics were conducted for the main predictors included in the regression models. The results showed that tolerance values for HNSRIL, ITCP, HWP, and the ITCP × HWP interaction term ranged from 0.591 to 0.748, and VIF values ranged from 1.336 to 1.692, suggesting that multicollinearity was not a substantial concern in the present study.

### 5.5 Hypothesis testing

Detailed regression estimates, including unstandardized coefficients (B), standard errors (SEs), and 95% confidence intervals (CIs), are provided in Supplementary Table S1.

#### 5.5.1 Association between head nurses’ scientific research and innovation leadership and nurses’ innovative work behavior

According to the hierarchical regression analysis presented in Model 3 of Table 3, after controlling for gender, age, and years of working, head nurses’ scientific research and innovation leadership was positively associated with nurses’ innovative work behavior (*β* = 0.488, *p* < 0.001). This finding supports Hypothesis 1.

**Table 3:** Hierarchical regression analysis.

Independent variable	ITCP		NIB		
	Model 1	Model 2	Model 3	Model 4	Model 5
Gender	0.043	0.009	0.129***	0.127***	0.132***
Age	−0.023	−0.061	0.223*	0.241**	0.223*
Years of working	0.132	0.092	0.011	−0.015	−0.009
Education level	0.135 **	0.045	0.090*	0.077*	0.081*
HNSRIL		0.520***	0.488***	0.339***	0.235***
ITCP				0.287***	0.263***
ITCP × HWP					−0.241***
*R^2^*	0.033	0.289	0.374	0.432	0.476
*△R^2^*	0.033	0.256	0.374	0.059	0.043
*F*	3.376 **	31.993***	47.020***	49.874***	50.800***

Values are standardized regression coefficients (β). Detailed regression estimates, including unstandardized coefficients (B), standard errors (SEs), and 95% confidence intervals (CIs), are provided in Supplementary Table S1. *p < 0.05, **p < 0.01, ***p < 0.001.

#### 5.5.2 The mediating effect of intra-team competition perception

Hierarchical regression analysis showed that head nurses’ scientific research and innovation leadership was positively associated with intra-team competition perception, as indicated in Models 2 and 4 of Table 3 (*β* = 0.520, *p* < 0.001). In turn, intra-team competition perception was positively related to nurses’ innovative work behavior (*β* = 0.287, *p* < 0.001). To further test Hypothesis 2, a bootstrap analysis with 5,000 resamples was performed. The results showed a significant indirect effect of head nurses’ scientific research and innovation leadership on nurses’ innovative work behavior through intra-team competition perception (effect = 0.158, 95% CI: 0.104, 0.214). Since the confidence interval did not include zero, intra-team competition perception was found to statistically mediate the association between head nurses’ scientific research and innovation leadership and nurses’ innovative work behavior, thus supporting Hypothesis 2.

#### 5.5.3 The moderating effect of harmonious work passion

The hierarchical regression results shown in Model 5 of Table 3 indicated that the interaction term between intra-team competition perception and harmonious work passion was significantly negatively associated with nurses’ innovative work behavior (*β* = −0.241, *p* < 0.001) suggesting that harmonious work passion moderated this relationship. To further probe the nature of the interaction, simple slope analyses were performed using PROCESS 4.1, with high and low harmonious work passion defined as one standard deviation above and below the mean. The findings revealed that intra-team competition perception was significantly associated with nurses’ innovative work behavior when harmonious work passion was low (*β* = 0.369, *p* < 0.001), whereas this association became non-significant when harmonious work passion was high (*β* = 0.011, *p* = 0.787). These results suggest that harmonious work passion attenuates the positive association between intra-team competition perception and nurses’ innovative work behavior, thereby supporting Hypothesis 3.

#### 5.5.4 The moderated mediation effect of harmonious work passion

**Figure 2:**
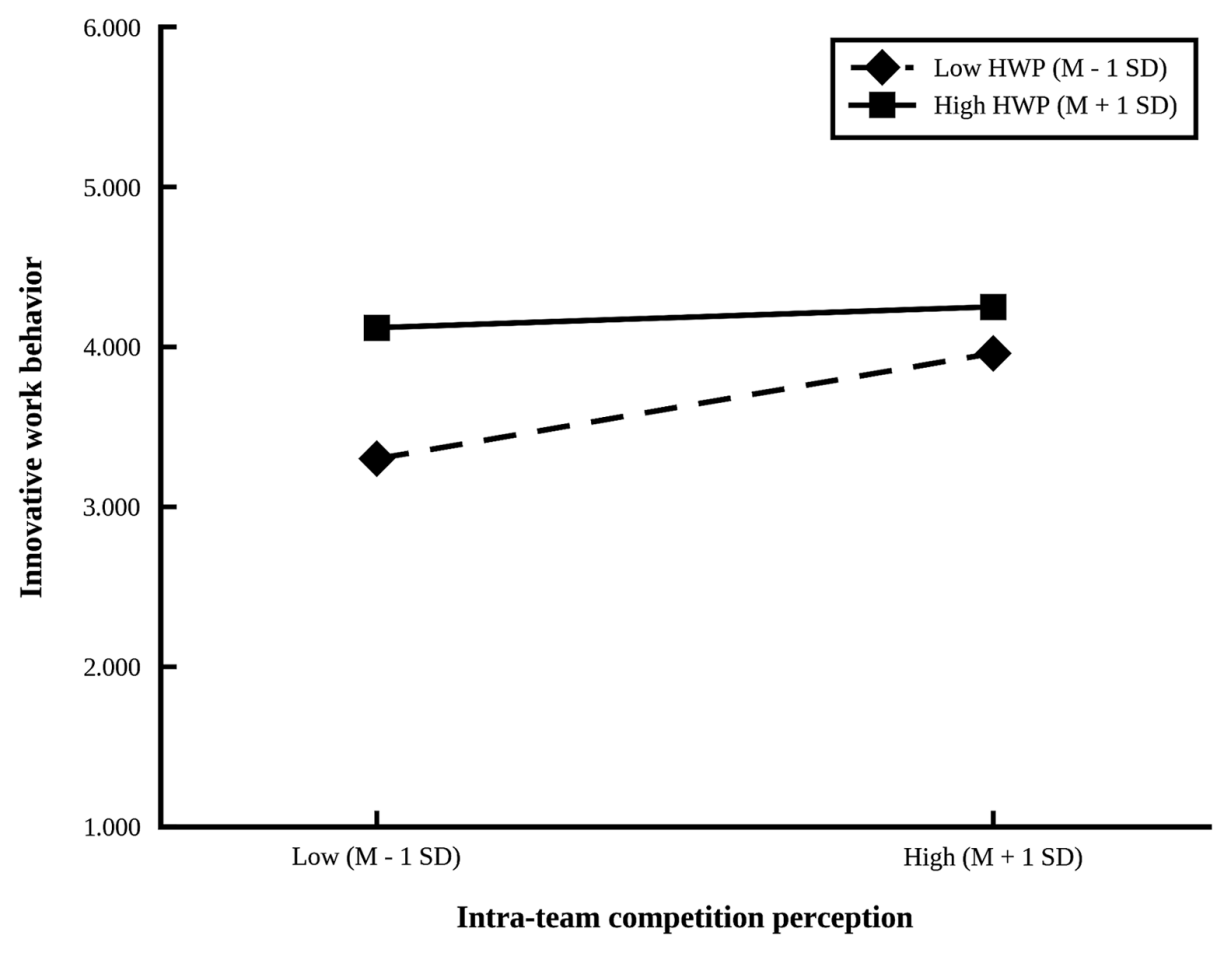
Moderating role of harmonious work passion in the association between intra-team competition perception and nurses’ innovative work behavior.

**Figure 3:**
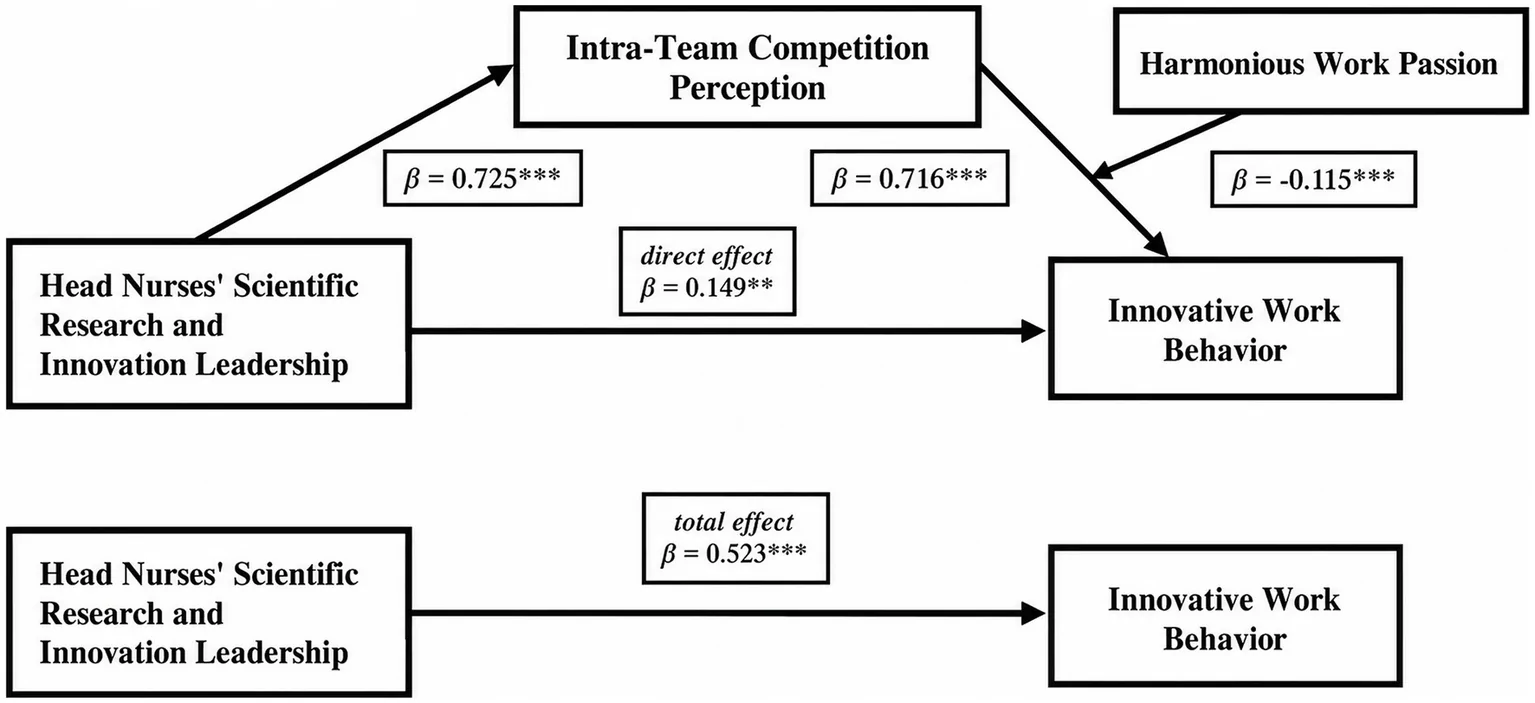
Moderated mediation model linking head nurses’ scientific research and innovation leadership to nurses’ innovative work behavior.

As illustrated in Figure 2, harmonious work passion moderated the indirect effect of head nurses’ scientific research and innovation leadership on nurses’ innovative work behavior via intra-team competition perception. The conditional indirect effects reported in Table 4 showed that this mediating pathway differed by levels of harmonious work passion. At low levels of harmonious work passion, the indirect effect was positive and statistically significant (effect = 0.268, 95% CI [0.196, 0.340]). At high levels of harmonious work passion, the indirect effect remained statistically significant, but its magnitude was notably smaller (effect = 0.079, 95% CI [0.036, 0.124]). Furthermore, the index of moderated mediation was significant (difference = −0.189, 95% CI [−0.284, −0.094]), indicating that the mediating role of intra-team competition perception was conditional on harmonious work passion. Taken together, these findings provide support for Hypothesis 4. Figure 3 presents the full conceptual model linking head nurses’ scientific research and innovation leadership to nurses’ innovative work behavior.

**Table 4:** Bootstrap tests for assessing the moderating effect of Harmonious Work Passion across different pathways.

Pathway	Effect	Indirect effect size	SE	Boot LLCI	Boot ULCI	Test result
HNSRIL → ITCP → NIB	Low HWP (−1 SD)	0.268	0.037	0.196	0.340	Sig.
	High HWP (+1 SD)	0.079	0.023	0.036	0.124	Sig.
	Difference in indirect effects	−0.189	0.048	−0.284	−0.094	Sig.

HNSRIL, Head nurses’ scientific research and innovation leadership; ITCP, Intra-team competition perception; HWP, Harmonious work passion; NIB, nurses’ innovative work behavior; Sig., significance.

## 6 Discussion

The results showed that head nurses’ scientific research and innovation leadership was positively associated with nurses’ innovative work behavior and was also indirectly associated with such behavior through intra-team competition perception. Moreover, harmonious work passion moderated the association between intra-team competition perception and nurses’ innovative work behavior, with the indirect association being stronger at lower levels of harmonious work passion and weaker at higher levels. These findings suggest that nurses’ innovative work behavior is related not only to leadership support, but also to the joint roles of team competition cues and harmonious work passion.

First, nurses’ innovative work behavior was found to be at a moderately high level in this study, with a mean score of 3.84 ± 0.83, suggesting that nurses possessed a certain foundation of innovative intention and participation. Among the dimensions, “idea generation” had the highest score, whereas “idea implementation” had the lowest score, indicating that nurses were more likely to identify problems and propose improvement ideas than to translate these ideas into practical outcomes (Zappalà et al., 2021). One possible reason is that idea generation depends more on individuals’ problem awareness and motivation for improvement, whereas innovation implementation usually requires higher-level conditions, such as time, resources, cross-departmental collaboration, and organizational support (Liehr and Hauff, 2024). It is noteworthy that the score for nurses’ innovative work behavior in this study exceeded that reported by Liu et al. (2026) in a random survey conducted in two tertiary hospitals in Henan Province, China (3.17 ± 0.72). This difference may be related to the relatively high proportion of participants with a bachelor’s degree. A higher educational background may help enhance nurses’ abilities in problem analysis and critical thinking (Urhan et al., 2022), while innovation incentive policies and experience in innovation teams may further strengthen their sense of participation, support, and responsibility in innovation activities, thereby improving the overall level of innovative behavior (Huang et al., 2022).

Second, Intra-team competition perception, conceptualized as a team-based motivational cognition, partially mediated the association between head nurses’ scientific research and innovation leadership and nurses’ innovative work behavior. In this study, intra-team competition perception was at a relatively high level (3.72 ± 1.10), and nurses with a master’s degree scored higher on this variable. This may be attributed to the fact that nurses with higher levels of education may be more likely to be perceive disparities in resource allocation and performance rewards (Nemati-Vakilabad et al., 2024; Song et al., 2024). Conceptually, stronger head nurses’ scientific research and innovation leadership may provide a context in which innovation-related activities and expectations are more emphasized within nursing teams (K. Liu et al., 2025). In such a context, nurses are more likely to perceive comparative signals of team competition related to innovation and interpret them as task-oriented competition centered on professional development and goal accomplishment, rather than as purely hostile competition (Lecic et al., 2023). This type of competition perception may be associated with nurses’ engagement in innovative work behavior within an innovation-oriented team context (O’Hara et al., 2022). It is important to note that this study does not suggest that competition is always beneficial for innovation. Rather, the findings indicate that intra-team competition perception may function as a constructive motivational signal when it is embedded within an innovation-oriented leadership context. Conversely, competition characterized by hostility, excessive interpersonal comparison, or resource exclusion may increase interpersonal conflict, reduce knowledge sharing, and undermine collaboration (Černe et al., 2014; Fletcher et al., 2008). Therefore, the positive association identified in this study should be interpreted as reflecting competition perceptions shaped by supportive and innovation-oriented leadership rather than competition itself. However, if competition evolves into hostile forms such as excessive comparison, resource exclusion, or interpersonal confrontation, it may damage the collaborative climate and hinder the sustainable development of innovative behavior (Yıldız et al., 2023).

Third, this study found that nurses’ harmonious work passion was relatively high (4.86 ± 1.40). Notably, nurses with longer professional experience in their profession, and those with a master’s degree exhibited markedly elevated levels of harmonious work passion in comparison to other demographics. Previous studies that a stronger sense of professional identity and self-efficacy is usually possessed by individuals with a higher harmonious work passion, and that stable intrinsic satisfaction from the work itself is more likely to be derived by them (Gkorezis et al., 2021). Therefore, the innovative behavior of these nurses is more likely to be driven by intrinsic motivation (Evli and Su Topbaş, 2025; Gkorezis et al., 2021). From this perspective, harmonious work passion may constitute a relatively stable internal motivational mechanism, thereby partially substituting for the motivating effect of team competition cues on innovative behavior (Cheyroux et al., 2025). Specifically, at high levels of harmonious work passion, the association between intra-team competition perception and nurses’ innovative work behavior was weaker; in contrast, at low levels of harmonious work passion, nurses’ innovation engagement may be more closely linked to external comparison and competition cues, making this pathway more salient. Thus, high harmonious work passion does not mean that nurses engage in less innovative behavior; rather, it means that their innovation engagement has greater intrinsic stability and is less likely to fluctuate substantially with changes in the external competitive environment. Compared with relying solely on competitive stimulation, cultivating nurses’ stable intrinsic work passion may be more conducive to promoting sustained innovation engagement (Gillet et al., 2025).

Finally, this study’s findings indicate that nursing managers should consider more than just the short-term incentives that come with competition when promoting nurses’ innovative work behavior. They should also place greater emphasis on creating conditions that support innovation and nurturing intrinsic motivation. On the one hand, head nurses’ scientific research and innovation leadership was positively associated with nurses’ innovative work behavior, suggesting that managers should strengthen head nurses’ capacities in scientific research guidance, resource coordination, and innovation support. On the other hand, the mediating role of intra-team competition perception suggests that perceptions of competition within teams may be associated with nurses’ innovative work behavior in the context of head nurses’ scientific research and innovation leadership. But excessive competition may undermine team collaboration and knowledge sharing, which is detrimental to the sustainable development of innovative behavior. In addition, the moderating role of harmonious work passion indicates that the sustained occurrence of nurses’ innovative work behavior cannot rely solely on external competitive stimulation, but needs to be grounded in identification with work and proactive engagement. Therefore, in nursing management practice, scientific research training, positive feedback, and career development support should be used to strengthen nurses’ sense of identification with and initiative toward scientific research and innovation work.

## 7 Limitations

This study has several limitations. First, the cross-sectional design limits causal interpretation and does not allow the temporal stability of the moderated mediation model to be examined. Future longitudinal or experimental studies are needed to further clarify the directionality of the observed associations. Second, all variables were measured using self-report questionnaires, which may introduce response bias and common method bias. Although Harman’ s single-factor test was conducted as a preliminary assessment, this approach alone cannot completely eliminate the possibility of common method variance. Future research could further address this issue by adopting more rigorous approaches, such as marker variable techniques, CFA marker models, or common latent factor approaches, as well as collecting data from multiple sources or using longitudinal designs. In addition, participants were recruited exclusively from tertiary hospitals in China, which may limit the generalizability of the findings to nurses in other cultural, healthcare, or organizational contexts. Moreover, although online data collection facilitated efficient recruitment, it may have introduced potential self-selection bias because nurses with greater interest in innovation-related issues may have been more likely to participate. Third, this study focused on head nurses’ scientific research and innovation leadership. Future research could compare different leadership styles and examine whether they are associated with nurses’ innovative work behavior through different psychological and team-based mechanisms.

## 8 Conclusion

This study showed that head nurses’ scientific research and innovation leadership was positively associated with nurses’ innovative work behavior and was indirectly associated with such behavior through intra-team competition perception. Harmonious work passion weakened the association between intra-team competition perception and innovative work behavior, indicating that nurses with stronger intrinsic motivation appeared less dependent on external competition cues. By integrating transformational leadership theory and self-determination theory, these findings extend existing research on nurses’ innovative work behavior by revealing a moderated mediation mechanism through which leadership is associated with innovation through team-level cognitive processes and individual motivational characteristics. This study highlights that nurses’ innovative behavior is shaped not only by leadership influences but also by the interplay between team-based social cognition and intrinsic motivational resources.

## Data Availability

The raw data supporting the conclusions of this article will be made available by the authors, without undue reservation.
