# Supplementary material for: Head nurses’ scientific research and innovation leadership and nurses’ innovative work behavior: the mediating role of intra-team competition perception and the moderating role of harmonious work passion
Source: Front Psychol. 2026 Sep 9;17:1900030. doi: 10.3389/fpsyg.2026.1900030 (PMC13597439; doi:10.3389/fpsyg.2026.1900030)
Supplement: Supplementary file 1 [file Table_1.DOCX]

Supplementary Table S1. Standard errors and 95% confidence intervals for the unstandardized regression coefficients in the hierarchical regression analyses.

| **Predictors** | **ITCP** | | **NIB** | | |
| --- | --- | --- | --- | --- | --- |
|  | **Model 1** | **Model 2** | **Model 3** | **Model 4** | **Model 5** |
| Gender | 0.425  [−0.467,1.205] | 0.366  [−0.644,0.794] | 0.867  [1.081, 4.490] | 0.827  [1.106, 4.356] | 0.796  [1.278, 4.406] |
| Age | 0.043  [−0.092, 0.075] | 0.037  [−0.094,0.050] | 0.087  [0.031, 0.372] | 0.083  [0.055, 0.380] | 0.080  [0.044, 0.357] |
| Years of working | 0.064  [−0.055, 0.196] | 0.055  [−0.058,0.157] | 0.130  [−0.240, 0.270] | 0.124  [−0.264, 0.222] | 0.119  [−0.246, 0.223] |
| Education level | 0.308  [0.236, 1.447] | 0.269  [−0.250,0.807] | 0.637  [0.161, 2.665] | 0.608  [0.016, 2.407] | 0.585  [0.116, 2.417] |
| HNSRIL |  | 0.012 [0.119,0.166] | 0.028  [0.283, 0.395] | 0.032  [0.173, 0.297] | 0.033  [0.098, 0.227] |
| ITCP |  |  |  | 0.114  [0.501, 0.948] | 0.110  [0.447, 0.879] |
| ITCP × HWP |  |  |  |  | 0.009  [−0.072, −0.035] |

Note. *N* = 400. Values outside square brackets are standard errors (SEs), and values within square brackets are 95% confidence intervals (CIs) for the unstandardized regression coefficients (B). HNSRIL = head nurses’ scientific research and innovation leadership; ITCP = intra-team competition perception; HWP = harmonious work passion; NIB = nurses’ innovative behavior. A dash indicates that the predictor was not entered into the corresponding model.
